# SAFETY OF PUBLIC BUSES IN NATURALLY OCCURRING RETIREMENT COMMUNITIES

**DOI:** 10.1093/geroni/igae098.4323

**Published:** 2024-12-31

**Authors:** Tracy Chippendale, Ruheena Sangrar, Shlomit Rotenberg, Angela Curl, Lisa Suzuki

**Affiliations:** New York University, New York, New York, United States; University of Toronto, Toronto, Ontario, Canada; University of Toronto, Toronto, Ontario, Canada; University of Otago, Christchurch, Otago, New Zealand; New York University, New York, New York, United States

## Abstract

Community mobility is critical to support aging-in-place. For older adults living in low-and moderate-income urban communities, driving may not be an option due to costs associated with car ownership, impracticalities of cars in urban settings, or a lack of driving history. Some older adults must retire from driving due to changes in their functional status. Safe public transportation is therefore paramount to equity in community mobility. The purpose of this Ecological theory informed study was to explore supports and limitations regarding safe public bus use in naturally occurring retirement communities (NORC). A multiple qualitative case study design was used with purposive sampling of representative cases, including four bus routes in NORC neighborhoods, two in Toronto, Canada, and two in NYC. Field observations and in-depth semi-structed interviews were conducted. Inclusion criteria for participants were: 1) Age 60 and older 2) Resident of one of the four NORC neighborhoods 3) Ride the bus on average 2 or more times per month 4) Ambulatory with or without a walking aid (e.g. cane, rollator) and 5) English speaking. A descriptive, thematic approach was used for data coding and analyses, and data was organized according to the Framework Method. Approximately 45 hours of field observations were conducted, and N=17 NORC residents participated in interviews. Results revealed four major themes: Impact of other people’s behavior, Problematic passageways: obstructions, Personal impact on safety, and Space & time orientation. Several subthemes also emerged. Results suggest implications for policy and education to increase safety for older adult bus users.

